# Effect of ozonated oil versus chlorhexidine gel among Indian patients with chronic periodontitis

**DOI:** 10.6026/973206300200837

**Published:** 2024-08-31

**Authors:** Avishek Singh, Naganandini S, Chhabra Kumar G, Chaudhary Pankaj, Dangi Shweta, Srivastava Harshit

**Affiliations:** 1 NIMS Dental College & Hospital, Jaipur, Rajasthan, India

**Keywords:** Chronic periodontitis, ozone oil, antimicrobial therapy

## Abstract

The efficacy of Ozonated oil in treating chronic periodontitis compared to Chlorhexidine gel following Scaling and Root Planing (SRP) is of interest. Fifty-six patients were randomly assigned to two groups: one receiving Ozonated oil post-SRP and the other receiving Chlorhexidine gel. Periodontal indices (Plaque Index, Gingival Index), Periodontal Probing Depth (PPD), and Clinical Attachment Level (CAL) were measured at baseline, one month, and three months. Intragroup comparisons for Plaque and Gingival indices showed statistically significant improvements over time (p < 0.05), but intergroup comparisons did not reveal significant differences. However, both groups demonstrated statistically significant improvements in PPD and CAL at all-time points (p < 0.05). Further research is warranted to explore Ozonated oil's potential as an antimicrobial treatment for chronic periodontitis and other oral microbial infections.

## Background:

Chronic periodontitis is a common oral disease among the adult population, characterized as an inflammatory condition affecting the tissues that support teeth, brought on by certain microorganisms. It can induce gingival recession, periodontal pocket formation, or progressive deterioration of the alveolar bone and periodontal ligament [1]. One of the best non-surgical treatments available is Scaling and Root Planing (SRP), which lowers the bacterial load in addition to reducing dental calculus and plaque [2]. Antimicrobial treatments must be used in addition to mechanical debridement in places that are difficult to access, such as deep pockets and furcation areas. A popular and efficient antibacterial agent used as a supplement after SRP is chlorhexidine (CHX). The material has a good substantively and exhibits effective bactericidal activity against different strains of microorganisms (gram positive and negative) at high concentrations. It should be mentioned moreover, that CHX shows greater cytotoxic effects [3-4]. Antimicrobial drugs can disturb eubiosis foster the perfect conditions for the colonization of pathogenic microorganisms and subsequent infections, potentially leading to recurring episodes [5-6]. Natural products like oils on the other hand have many chemically active compounds having good anti-microbial activity. It has been shown; meanwhile, that adding ozone to oils might improve their qualities even further. In fact, ozone (O3) is being used as a clinical agent in many microbial diseases and is acknowledged as a potent antiviral, anti-bacterial and antifungal agent [7- 8]. The underlying processes of this action stem from ozone's stronger oxidizing characteristics, which causes disruption or break in the continuity of cell wall and the cytoplasmic membrane resulting in an increased permeability and ozone penetration into bacterial cells are the outcomes [9]. In addition, ozone has the power to amplify the innate immune system's defense against microbes. Ozone has gained popularity in several therapeutic procedures developed recently to suppress infectious microorganisms in tooth plaque and treat dental infections linked to periodontal diseases as well as many other oral diseases [10-11]. However, there is a scarcity of literature showing effects of ozone as a potent antimicrobial agent. Therefore, it is of interest to show the effect of ozonated oil in patients with chronic periodontitis against chlorhexidine gel after scaling and root planning.

## Materials & Methods:

## Study design and setting:

A randomized controlled trial was conducted in the NIMS Dental College and Hospital, Rajasthan in which 56 patients (calculated using G Power software version 3.1.9.7 with effect size=0.68, α error =0.05, Power 1-β =0.80, allocation ratio N2/N1=1) with chronic periodontitis were included. Study was conducted from June 2022 to June 2023. Study design and methodology was presented in front of the institutional ethical committee board members of NIMS University and ethical approval was obtained before starting the study. Reference number of the study was NIMSUR/IEC/2022/298 and Proposal number is IEC/P-32/2022. All clinical procedures and data collection was done in the Department of Public Health Dentistry, NIMS Dental College and Hospital, Rajasthan. All the procedures followed were in accordance with the ethical standards of the institutional ethical committee and with the Helsinki Declaration of 1975, as revised in 2000.

## Inclusion and exclusion criteria:

Patients suffering from chronic periodontitis with a minimum of at least 10 teeth present in each arch, gingival bleeding on probing present, periodontal pocket depth of at least three mm and clinical attachment level of more than two mm were included in the study. Whereas patients with a history of any systemic disease such as diabetes, hypertension, anemia and any systemic disease related to periodontitis, patients with fixed prosthesis, removable prosthesis or any orthodontic appliance, history of pregnancy or breastfeeding, history of use of systemic drugs in last 3 months and patients who have undergone SRP in the last 6 months were excluded from the study.

## Study procedure:

A consent form was duly signed by the patient after explanation of the study purpose and methodology before the commencement of the study, 56 Patients were randomly divided in two groups (one and two). Follow up period of the study was three months. A detailed case history was recorded in a specially designed performa and Plaque index (PI) measured using Turesky- Gilmore - Glickman modification of the Quigley-Hein Plaque index (1970), Gingival Index (GI) measured using Loe H and Sillness J Gingival index (1963), Periodontal probing depth (PPD) measured using Williams Periodontal probe (distance measured from the gingival margin to the pocket base) and Clinical attachment level (CAL) (measured from cemento-enamel junction to the base of pocket) were measured at a time interval of baseline, one month and three month. For group one complete oral prophylaxis was done and patients were advised to apply Ozone oil daily for a period of one month and for group two complete oral prophylaxes were done and patients were advised to apply chlorhexidine gel daily for a period of one month. Two operators conducted oral procedures and outcomes assessment.

## Blinding:

Blinding the treatment-giving operator wasn't possible, but they had no interaction with other researchers. Neither operator knew the participants' treatments. Data assessors and analysts remained blinded throughout the trial, with assessors instructed not to learn individual treatments from patients.

## Outcome assessment:

All the data was collected at baseline, one month and three month interval after randomization. Primary outcome was to check whether there is any difference in the clinical parameters measured before and after the use of ozone oil and secondary outcome was to check the effectiveness of ozone oil when compared to chlorhexidine gel in the treatment of chronic periodontitis after SRP.

## Statistical analysis:

All data of baseline and recall examination was entered into Microsoft Office Excel and transferred into SPSS Software for further statistical analysis. Mean and standard deviation was calculated. Unpaired t-Test was applied for intergroup differences and paired t test was used for intragroup comparisons. P value <0.05 was considered statistically significant.

## Results:

A total of 56 patients (Table 1) were selected after satisfying inclusion and exclusion criteria and thorough clinical examination was done to record the required clinical parameters (modified PI, GI, periodontal probing depth and loss of attachment measured by CAL). Significant difference was seen in the plaque scores and gingival scores from baseline to one month and three months but intergroup differences were not significant (Table 2 & Table 3). There was an improvement in all parameters in both ozone and chlorhexidine group. In periodontal probing depth and clinical attachment level significant intragroup difference was seen from baseline to three months follow up but in PPD significant intergroup was seen at three months follow up (Table 4) and CAL significant intergroup difference was seen at one month and three months follow up (Table 5).

## Discussion:

Different bacterial accumulation over the soft tissues supporting the tooth is the cause of both the onset and progression of periodontitis. Mechanical SRP is a routine method of reducing the number of pathogenic microbes, which is necessary to control the disease prevalence and severity. A study by Meseli *et al.* [12] found that attachment loss, as opposed to gain, occurs at the sites with PPD of three mm after SRP. Antimicrobial drugs are used as an adjuvant to SRP in order to facilitate microbial destruction [13]. Numerous antibacterial medicines, including tetracycline and CHX, are available; however, each drug has a specific side effect. Extrinsic tooth discoloration is the most typical negative effect linked to CHX use. This can be explained by a local precipitation interaction between chromogens present in food and drink and tooth-bound chlorhexidine [14]. Ozone oil, which works by altering the subgingival environment, provides an alternative to traditional antibacterial treatments. There are multiple methods of supplying ozone; however, it cannot be preserved since it breaks down quickly into a complicated web of chain reactions when it dissolves in water. Its life period increases in years when it is dissolved in an oil base. Ozonated oil was therefore used for this investigation rather than ozonated water because it has been reported that oil application resulted in a prolonged duration in the oral cavity, sufficient medication penetration, excellent efficacy, and acceptance.

The study's findings demonstrate a significant improvement in intragroup scores, relative to the baseline, for all clinical indices assessed. Furthermore, in contrast to intergroup differences, intragroup differences were often substantial, which was consistent with research by Gandhi *et al.* [15], Colombo *et al.* [16], and Shruti *et al.* [17]. The potential reason for the improvement in all clinical indices after SRP treatment and ozone oil treatments could be attributed to the antibacterial properties of the latter, which act as an oxidant. If taken in small doses, this chemical molecule can also boost the immune system, release growth hormones, alter blood vessels and hematopoiesis, and activate local antioxidant systems. On the other hand, concentrating on the notable improvement in PPD and CAL, this can be attributed to both increased angiogenesis with gingival tissue revascularization and connective tissue repair, which is attributed to ozone's stimulating effect on fibroblasts [18]. Between the intergroup values of PI and GI at baseline, one month, and three months, there is no discernible difference. In research by AL-Chalabi *et al.* [19] gingival crevicular fluid volume and interleukin-1β concentration were shown to be significantly lower in the ozone oil-treated group when CHX or ozonated oil was administered to the gingiva of patients with plaque-induced gingivitis. This emphasized the data supporting the ozone oil. Other studies conducted by Shruti Lendhey *et al.* Marco Colombo *et al.* Kaveri *et al.* also found that though there was a significant reduction in PI and GI within a group but when the experimental group was compared with control group there was no significant difference i.e. Ozone oil was as effective in reducing Plaque and gingivitis as CHX. At the end of the three-month period, PPD had significantly decreased from baseline, and the ozone oil group's reductions from baseline to three months were much greater than those of the CHX group. This result was in contradiction with the study by Gandhi *et al.* Clinical attachment level also showed significant inter group difference. It was found that Ozone group had significant improvement in CAL as compared to CHX group. The significance in difference can be seen both at one month and three months. This was in contradiction to study conducted by Colombo *et al.* [16], and Gandhi *et al.* Contrary to what has been stated above, Pietrocola *et al.* [20] evaluated the properties of a new ozonated oil (ozone oil) against pathogens causing oral disease especially periodontal disease and compared it with that of chlorhexidine based antimicrobial agent. They discovered that the ozonized olive oil has less antibacterial activity than the CHX-based agents tested. Additionally, Uraz *et al.* [21] revealed that adjunctive ozone therapy did not improve clinical, microbiological, and biochemical parameters above SRP in patients with chronic periodontitis. On the other hand, a study done by Shruthi Nambiar *et al.* [22] reported that ozonated oil could enhance the outcomes of SRP for the treatment of periodontal diseases. Based on available data, ozone exhibits antibacterial properties and a high degree of biocompatibility with both gingival fibroblasts and periodontal cells. The study's findings indicate that, while not having a bigger impact than the conventional SRP plus chlorhexidine combination, the application of the ozonized oil for the non-surgical therapy of periodontal disease constitutes a viable strategy. This study provides valuable insights on the anti-microbial effect of ozonated oil, Furthermore; its use can also be explored for the treatment of other oral diseases like ulcers, candidiasis, dry socket and others.

## Limitations:

One of the major limitations of this study was that it was done solely in a clinical manner. Only clinical indices were measured and microbiological data was not collected. The effect of ozonated oil on the periodontal microbial flora would be an interesting area which should be subjected to further research. Another limitation of this study was that it was only for duration of 3 months and a sample size of only 56 was included. Further research on a larger population and for a longer duration is recommended. 

## Conclusions:

Ozonated oil, when used adjunctively with SRP, shows significant improvements in clinical parameters of chronic periodontitis comparable to Chlorhexidine gel. It demonstrates superior efficacy in reducing periodontal pocket depth and attachment loss compared to Chlorhexidine. This suggests ozonated oil as a promising, cost-effective, and non-invasive homecare option for enhancing gingival and periodontal health.

## Figures and Tables

**Table 1:** Demographic details of study participants

**Study Group**	**N**	**Mean Age**	**Gender**	
			**Males**	**Females**
Group 1 (Ozone + SRP)	28	45.18	21	7
Group 2 (Chlorhexidine + SRP)	28	45.21	21	7

**Table 2:** Comparison of mean value of plaque score

**Groups**	**Baseline**	**1 Month**	**3 Month**	**p value**
Ozonated oil	4.44± 0.30	2.57± 0.58	3.31±0.38	Baseline & 1 month <0.001*
Chlorhexidine	4.40±0.20	2.62±0.64	3.31±0.55	Baseline & 3months <0.001*
Difference	0.03	0.08	0.1	Baseline & 1 month <0.001*
P value	0.51 NS	0.74 NS	0.98 NS	Baseline & 3months <0.001*
NS = not significant
* = significant (p value <0.05)

**Table 3:** Comparing mean value of gingival score in two groups

**Groups**	**Baseline**	**1 Month**	**3 Month**	**p value**
Ozonated oil	2.22±0.22	1.18±0.15	1.53±0.27	Baseline & 1 month <0.001*
Chlorhexidine	2.11±0.26	1.18±0.27	1.55±0.28	Baseline & 3months <0.001*
Difference	0.1	0.002	0.02	Baseline & 1 month <0.001*
P value	0.117 NS	0.962 NS	0.738 NS	Baseline & 3months <0.001*
NS = not significant
* = significant (p value <0.05)

**Table 4:** Comparing mean value of periodontal probing depth in two groups

**Groups**	**Baseline**	**1 Month**	**3 Month**	**p value**
Ozonated oil	5.21±0.58	3.04±0.76	3.78±0.36	Baseline & 1 month <0.001*
Chlorhexidine	5.28±0.35	3.10±0.58	4.04±0.56	Baseline & 3months <0.001*
Difference	0.07	0.06	0.26	Baseline & 1 month <0.001*
P value	0.68 NS	0.63 NS	<0.001 *	Baseline & 3 months <0.001*
NS = not significant
* = significant (p value <0.05)

**Table 5:** Comparing mean value of clinical attachment level in two groups

**Groups**	**Baseline**	**1 Month**	**3 Month**	**p value**
Ozonated oil	6.32±0.46	4.14±0.68	4.76±0.47	Baseline & 1 month <0.001*
Chlorhexidine	6.38±0.33	4.32±0.57	5.02±0.32	Baseline & 3months <0.001*
Difference	0.06	0.18	0.26	Baseline & 1 month <0.001*
P value	0.52 NS	0.03*	<0.001*	Baseline & 3months <0.001*
NS = not significant;
* = significant (p value <0.05)

## References

[1] Newmann MG (2002). Clinical Periodontology..

[2] Berezow AB, Darveau RP (2011). Periodontology 2000.

[3] Liu JX (2018). J Bone Jt Infect..

[4] Coelho AS (2020). Odontology..

[5] Mattappalil A, Mergenhagen KA (2014). Clin Ther..

[6] Lagacé-Wiens P, Rubinstein E (2012). Expert Opin Drug Saf..

[7] Martínez-Sánchez G (2005). Eur J Pharmacol..

[8] Smith NL (2017). Med Gas Res..

[9] Nagayoshi M (2004). Oral Microbiol Immunol..

[10] Aerts O (2016). Contact Dermatitis..

[11] Kumar T (2016). Contemp Clin Dent..

[12] Meseli SE (2017). J Istanb Univ Fac Dent..

[13] Drisko CL (1995). J Periodontol..

[14] Shoukheba MYM, ShA Ali (2014). Tanta Dental Journal.

[15] Gandhi KK (2019). BDJ Open..

[16] Colombo M (2021). Biology (Basel)..

[17] Lendhey SS (2020). Journal of Oral Research and Review..

[18] Ugazio E (2020). Molecules..

[19] AL-Chalabi MZ, Mohamed AN (2019). J. Pharm. Sci. & Res..

[20] Pietrocola G (2018). J Clin Exp Dent..

[21] Uraz A (2019). J Dent Sci..

[22] Nambiar S (2022). J Pharm Bioallied Sci..

